# Hybrid Treatment with Complete Transposition of Supra-Aortic Trunks *versus* Conventional Surgery for the Treatment of Aortic Arch Aneurysm

**DOI:** 10.21470/1678-9741-2017-0052

**Published:** 2017

**Authors:** Leonardo de Oliveira Souza, Rodrigo de Castro Bernardes, Túlio Pinho Navarro, Ricardo Jayme Procópio, Fernando Antônio Roquete Reis Filho, Luiz Claudio Moreira Lima, Ernesto Lentz da Silveira

**Affiliations:** 1 Hospital Márcio Cunha, Ipatinga, MG, Brazil.; 2 Hospital Madre Teresa, Belo Horizonte, MG, Brazil.; 3 Faculdade de Medicina da Universidade Federal de Minas Gerais (FM-UFMG), Belo Horizonte, MG, Brazil.; 4 Hospital das Clínicas da Universidade Federal de Minas Gerais (HC-UFMG), Belo Horizonte, MG, Brazil.

**Keywords:** Aortic Aneurysm, Aorta, Thoracic, Cardiovascular Surgical Procedures, Endovascular Procedures

## Abstract

**Objective:**

The disease of the aortic arch is traditionally approached by open surgical repair requiring cardiopulmonary bypass and circulatory arrest. This study performed a retrospective analysis comparing outcomes through primary hybrid patients submitted to aortic arch surgery without cardiopulmonary bypass with patients submitted to conventional open surgery.

**Methods:**

25 patients submitted to the aortic arch surgery were selected in the period 2003-2012 at the Madre Teresa Hospital in the city of Belo Horizonte, Brazil; 13 of these underwent hybrid technique without cardiopulmonary bypass and 12 underwent conventional open surgery.

**Results:**

The mortality rate for the hybrid group was 23% and for the conventional surgery group was 17% (*P*=0.248). The postoperative complication rate was also similar in both groups, with no significant difference.

**Conclusion:**

Both techniques proved to be similar in mortality and morbidity. However, due to the small sample, more analytical studies with larger samples and long-term follow-up are needed to clarify this issue.

**Table:** 

Abbreviations, acronyms & symbols				
ACT	= Activated clotting time		HF	= Heart failure
AF	= Atrial fibrillation		ICU	= Intensive care unit
AMI	= Acute myocardial infarction		MTH	= Madre Teresa Hospital
ARF	= Acute renal failure		REC	= Research Ethics Committee
COPD	= Chronic obstructive pulmonary disease		SAH	= Systemic arterial hypertension
CPB	= Cardiopulmonary bypass		TQT	= Tracheostomy
DM	= Diabetes mellitus		UFMG	= Federal University of Minas Gerais
ECC	= Extracorporeal circulation			

## INTRODUCTION

Treating patients with aortic arch aneurysm is a major technical challenge and it is an area in continuous development and innovation^[1]^.

The aortic arch aneurysm is a lethal disease^[2-5]^ that represents 10% of thoracic aneurysms^[3]^. The surgical treatment, indicated in aneurysms larger than 6 cm^[6,7]^, alters its natural course^[8]^. However, surgery requires cardiopulmonary bypass (CPB) with deep hypothermic and circulatory arrest^[1,9]^, with 65% to 80% of morbidity rates and mortality from 10% to 20% due to stroke, acute myocardial infarction (AMI), pulmonary complications and excessive bleeding^[1,6,10]^.

Novaes et al.^[3]^ developed an alternative technique with intraluminal ring that reduces the extracorporeal circulation (ECC) time and bleeding, but does not contribute to decreasing the overall mortality.

Hybrid techniques with transposition of the supra-aortic trunks associated with endovascular surgery^[11,12]^ prevents the need of cardiopulmonary bypass, which theoretically would reduce the morbidity and mortality^[13,14]^. Therefore, this would be an alternative option for high-risk patients^[1]^.

On the other hand, some other authors did not show better results. Schumacher et al.^[15]^ reported 20% of mortality within 30 days; Andersen et al.^[16]^ showed an increasing of the mortality rate (from 5.7% to 14.9%) after 30 days of hybrid treatment in Zone 0 mainly due to retrograde dissection, and Melissano et al.^[17]^ observed a stroke incidence of 14.3% in patients treated through the hybrid mode.

Thus, comparing the hybrid techniques and the conventional repair is necessary in order to establish their true impact on the overall morbidity and mortality.

The aim of this study is to compare both rates of mortality and postoperative complications between the hybrid technique and conventional surgery for treating patients with aortic arch aneurysm.

## METHODS

This is an observational, analytical and retrospective case-control study comparing conventional surgery (group 1) and the hybrid technique (group 2).

The study was approved by the research ethics committee (REC) the Madre Teresa Hospital (MTH), as well as by the REC-UFMG (number 306224). Between 2003 and 2012, in the MTH (Belo Horizonte, Brazil), from 948 patients submitted to aortic surgery in all its segments, 25 patients were selected for the treatment of aneurysmal disease exclusively in the aortic arch.

Conventional surgery was performed in 12 patients with cardiopulmonary bypass, deep hypothermic, and complete or partial circulatory arrest. Hybrid surgery was performed in 13 patients with full supra-aortic trunks transposition and aneurysm removal through stent.

### Inclusion Criteria

- Absence of concentric calcification of the ascending aorta and arch (porcelain aorta).
- Suitable diameter of the ascending aorta for proximal stent sealing - when either this diameter was larger than 42 mm or a type 1 intraoperative endoleak occurred, the banding technique with polyester prosthesis retail (Dacron^®^) was used.
- Normal aortic valve function.
- Good quality of the vessels for the stent's retrograde access (femoral and iliac arteries' diameter, tortuosity and calcification).

### Exclusion Criteria

- Aortic dissection.
- Aortic arch aneurysm involving also the ascending and/or descending aorta.
- Insufficient and/or moderate to severe aortic valve stenosis.
- Unfavorable anatomy of the femoral and iliac vessels for the thoracic stent navigation.
- Unstable patients with neurological condition without prognosis.

### Hybrid Surgery Technique

Transposition was performed at the ascending aorta, after median mini-sternotomy with polyester graft (Dacron^®^) for end-to-side anastomosis, and tangential clamping of the aorta in Zone 0 (Ishimaru's classification)^[14,18]^ about 3 cm above the sinotubular junction. It was carried out through intrathoracic access, with distal end-to-end anastomosis of the arteries left common carotid, innominate, and left subclavian, all at once without neck incision.

The stent's choosing, sizing, and implantation strategy was determined preoperatively after analyzing the 16-row multislice computed tomography scans with multiplanar reconstructions (center line included). The choice of the stent trademark was done according to each device's specific characteristics, such as warhead type (short or long), device's flexibility and diameter size (French).

### Conventional Surgery Technique

Subsequently to the median sternotomy and right axillary artery dissection, the following procedures were proceeded: pericardiotomy, supra-aortic trunks exposure, systemic heparin administration with the activated clotting time (ACT) control, right axillary artery cannulation, inferior vena cava cannulation across the right atrium, cardiac arrest induction throughout antegrade cold blood crystalloid cardioplegia, deep and moderate hypothermia, aortic arch replacement by polyester graft (Dacron^®^) and proximal end-to-end anastomosis in the ascending aorta, followed by supra-aortic trunks revascularization by intrathoracic access; using partial circulatory arrest (with selective cerebral flow through right axillary artery) or total, and distal end-to-end anastomosis in the descending aorta or its distal arch.

### Follow-up

Patients were evaluated by means of clinical examination; data collection of medical records and computerized tomography scans.

Data collection was performed through hospital's electronic-stored medical records investigation, comprising: laboratory tests and images either attached to the system or brought by the patients during the postoperative follow-up visits.

A standard form was utilized containing the following items: gender, age, admittance date, surgery date, surgery type (elective or emergency), symptoms, comorbidities, circulatory arrest, technical success, CPB time, stent trademark and endoleak occurrence, intensive care unit (ICU) time, hemoderivatives administrated, hospital discharge date, and death (early and late).

All collected data were anonymous and confidential.

Frequency distribution tables of categorical variables for the subjects' characterization were presented, as well as the central tendency and dispersion measures for continuous variables. To analyze the differences between groups, chi-square and Fisher's exact test were utilized for categorical variables and the Mann-Whitney test was used for continuous variables.

The primary outcomes were: hospitalization time, ICU time, postoperative complications, early and late death, and cause of death. It was considered significant *P*<0.05. Database processing and analyses were performed using the software Stata (version 12).

## RESULTS

Data of 18 men and 7 women were analyzed. The average age was 66 years. There were no significant differences in the demographic characteristics between the conventional technique and the hybrid technique, as presented in Table 1.

**Table 1:** Demographic characteristics distribution of patients submitted to aortic arch aneurysm repair between groups hybrid technique and conventional technique in a hospital in the city of Belo Horizonte, in the period 2003-2012.

Demographic characteristics	Hybrid technique		Conventional technique		Total		*P* value
	n	%	n	%	n	%	
Gender							
Male	8	61.54	10	83.33	18	72	0.225*
Female	5	38.46	2	16.67	7	28	
Age median (min.; max.)	69 (33;77)		61 (44;75)		66 (33;77)		0.134**

* Chi-square test.

** Wilcoxon test

Regarding the surgery type, no significant differences were observed between the groups submitted to hybrid surgery and conventional surgery, once the majority of operations were urgent (72%) in both groups.

Considering the symptoms, no significant difference was observed between the groups submitted to hybrid surgery or conventional surgery (Table 2).

**Table 2:** Presence and type of symptoms distribution of patients submitted to arch aneurysm repair in a hospital in the city of Belo Horizonte between groups hybrid surgery and conventional surgery, in the period 2003-2012.

Symptoms	Hybrid technique		Conventional technique		Total		*P* value
	n	%	n	%	n	%	
Asymptomatic	2	15.4	__	__	2	8	0.480*
Symptoms							
Chest pain	7	53.9	9	75	16	64	0.411*
Precordial pain	__	__	2	16.67	2	8	0.220*
Abdominal pain	__	__	2	16.67	2	8	0.220*
Interscapular pain	__	__	1	8.33	1	4	0.480*
Hoarseness	1	7.7	2	16.67	3	12	0.593*
Cervical tumor	__	__	1	8.33	1	4	0.480*
Dyspnea on mild exertion	2	15.4	1	8.33	3	12	1.000*
Superior vena cava compression syndrome	__	__	1	8.33	1	4	0.480*
Right hemiparesis	__	__	1	7.69	1	4	1.000*
Transient aphasia	1	7.7	__	__	1	4	1.000*
Syncope	1	7.7	__	__	1	4	1.000*
Hematemesis and hemodynamic instability	1	7.7	__	__	1	4	1.000*

* Fisher's exact test

In relation to the occurrence of comorbidities and evaluation, no significant differences were found between the groups (Table 3).

**Table 3:** Presence and type of comorbidities distribution of patients submitted to arch aneurysm repair in a hospital in the city of Belo Horizonte in the groups hybrid surgery and conventional surgery, in the period 2003-2012.

Comorbidities	Hybrid technique		Conventional technique		Total		*P *value
	n	%	n	%	n	%	
No comorbidities	1	7.69	__	__	1	4	1*
Comorbidities							
SAH	2	15.38	__	__	2	8	0.480*
COPD	3	23.08	2	16.67	5	20	1*
HF	1	7.69	1	8.33	2	8	1*
Former smoker	__	__	1	8.33	1	4	1*
Coronary insufficiency	1	7.69	1	8.33	2	8	1*
Prior stroke	1	7.69	1	8.33	2	8	1*
Dyslipidemia	1	7.69	__	__	1	4	1*
Obesity	3	23.08	__	__	3	12	0.220*
AF	1	7.69	__	__	1	4	1*
DM	1	7.69	__	__	1	4	1*

* Fisher's exact test. AF=atrial fibrillation; COPD=chronic obstructive pulmonary disease; DM=diabetes mellitus; HF=heart failure; SAH=systemic arterial hypertension

The technical success rate was 100%. In the hybrid group, endoleak occurred in 2 (15.3%) patients: one type-IA, corrected throughout an ascending aorta cerclage before surgery, and the other a type-IIB, that was followed up clinically with thorax computed tomography scanning and spontaneous remission after 6 months.

The thoracic stents used in patients (n=13) were: Braile^®^ in 1 (7.6%) patient, Evita^®^ in 3 (22.8%) patients, Gore^®^ in 1 (7.6%) patient, Medtronic^®^ in 6 (46.7%) patients and Zenith^®^ in 2 (15.3%) patients.

Regarding the conventional surgery group, circulatory arrest was partial in 7 (58.33%) patients, and total in 5 (41.67%) patients, with an average CPB time of 92.5 minutes.

Postoperative complications occurred in all patients who underwent conventional surgery. In the hybrid surgery group, 10 (77%) patients had postoperative complications.

The occurrence of complications was not significantly different between groups (Table 4).

**Table 4:** Presence and type of complications distribution of patients submitted to arch aneurysm repair in a hospital in the city of Belo Horizonte in the groups hybrid surgery and conventional surgery, in the period 2003-2012.

Complications	Hybrid technique		Conventional technique		Total		*P *value
	n	%	n	%	n	%	
No complications	3	23.08	-	-	3	12	0.220*
Complications							
Diffuse bleeding	1	7.69	3	25	4	16	0.322*
AMI	1	7.69	1	8.33	2	8	1*
Pleural effusion	3	23.08	4	33.33	7	28	0.673*
Stroke	2	15.38	2	16.67	4	16	1*
Mediastinitis	__	__	1	8.33	1	4	0.480*
Phrenic nerve injury	__	__	1	8.33	1	4	0.480*
Pneumothorax	1	7.69	1	8.33	2	8	1.000*
Hemodynamic instability	__	__	1	8.33	1	4	0.480*
Cardiogenic shock	__	__	1	8.33	1	4	0.480*
Pneumonia	3	23.08	2	16.67	5	20	1*
TQT	__	__	2	16.67	2	8	0.220*
Wound infection	__	__	1	9.09	1	4.17	0.458*
AF	2	15.38	1	8.33	3	12	1*
Sepsis	__	__	1	8.33	1	4	0.480*
Pericardial effusion	1	7.69	__	__	1	4	1*
Respiratory failure	1	7.69	__	__	1	4	1.000*
ARF	1	7.69	__	__	1	4	1.000*

* Fisher's exact test. AF=atrial fibrillation; AMI=acute myocardial infarction; ARF=acute renal failure; TQT=tracheostomy

There was no statistical difference between groups regarding in-hospital mortality (Table 5).

**Table 5:** In-hospital mortality in patients submitted to arch aneurysm repair in a hospital in the city of Belo Horizonte in the groups hybrid surgery and conventional surgery, in the period 2003-2012.

Deaths	Hybrid technique		Conventional technique		Total		*P* value
	n	%	n	%	n	%	
In-hospital	3	23	2	17	5	20%	0.248*

* Fisher's exact test

The most common causes of in-hospital deaths were diffuse hemorrhage and cardiogenic shock, with no statistical significant difference between groups (Table 6).

**Table 6:** Causes of in-hospital deaths in patients submitted to arch aneurysm repair in a hospital in the city of Belo Horizonte in the groups hybrid surgery and conventional surgery, in the period 2003-2012.

Causes of in-hospital deaths	Hybrid technique	Conventional technique
	n	n
Stroke	1	__
Cardiogenic shock	1	__
Diffuse bleeding	1	2
Total	3	2

The postoperative follow-up ranged from 39 to 51 months (mean 46 months). Four patients from the conventional surgery group went missing during follow-up: after 4 years and 2 months, 3 years and 9 months, 3 years and 3 months and 2 years and 7 months; these periods refer to their last medical visit, and telephone contact was tried with all of them, but unsuccessfully. In the conventional surgery group, one patient died of lung cancer after 3 years and 8 months after surgery and another one died after 3 years and 5 months due to extensive stroke.

With respect to post-hospital deaths in the hybrid surgery group, the causes were: bladder cancer 9 months after surgery; "indeterminate" 3 months after surgery (died in another hospital and the family said that he had just "got sick"), pneumonia after 74 days (readmitted at MTH) and AMI after 57 days (readmitted at MTH).

The ICU time was similar in both groups: 3 days in the hybrid technique group and 2 days in the conventional technique group with no significant difference between them (*P*=0.805). In relation to the hospitalization time, in days, the average was higher among patients submitted to the conventional technique (18 days); however, the difference was not statistically significant (Table 7).

**Table 7:** ICU and hospitalization times in patients submitted to arch aneurysm repair in a hospital in the city of Belo Horizonte in the groups hybrid surgery and conventional surgery, in the period 2003-2012.

	Hybrid technique	Conventional technique	Total	*P* value
	Median (min; max)	Median (min; max)	Median (min; max)	
ICU time (days)	3 (0; 16)	2 (0; 25)	3 (0; 25)	0.805
Hospitalization time (days)	8 (5; 70)	18 (3; 42)	17 (3; 17)	0.862

## DISCUSSION

In this case-control study (retrospective cohort), two groups were selected with similar demographic and clinical characteristics.

However, we did not know about the severity score of surgical risk for both groups, since these data were not available during the preparation of this study. It is likely that the more severe patients were allocated in the hybrid surgery group, which could explain the higher mortality rate found, although this was not statistically significant.

In the hybrid surgery group, in-hospital deaths were due to stroke, diffuse bleeding and cardiogenic shock, and three patients were taken to emergency surgery: the first patient died after 3 days of surgery by multiple organ failure following an extensive stroke; the second patient presented diffuse bleeding and died on the first postoperative day; the third patient presented a low cardiac debit due to left ventricular failure before surgery and died during surgery.

In the conventional surgery group, in-hospital death occurred due to diffuse bleeding in two patients, who were operated on an emergency basis and showed CPB time over 150 minutes. One patient died in the immediate postoperative period and the other one died in the second postoperative day.

The follow-up time was satisfactory (46 months). In the conventional surgery group, four patients were missed after about 3 years of follow-up. In this group there were two late deaths not related to aneurysm (lung cancer and cerebral ischemia). The other patients, until our last contact, were asymptomatic.

In the hybrid technique group was observed four posthospital deaths, three not directly related to the surgery or illness: one patient died because of bladder cancer, another one died due to bacterial pneumonia, and the third died due to acute myocardial infarction. One patient died three months after surgery due to undefined causes and may be caused by an aneurysm rupture.

The complication rates did not differ between groups. The stroke rate was 15.3% in the hybrid technique and 16.6% in the conventional technique. According Melissano et al.^[17]^, both the ascending aorta manipulation during clamping to transpose the supra-aortic vessels and the aorta's sealing to anchor the thoracic stent increase the stroke incidence rate up to 14.3%, which could explain this high rate in patients submitted to the hybrid technique.

Pulmonary complications, including pneumonia, pleural effusion and tracheostomy (TQT), did not statically differ, although TQT was observed only in the conventional surgery group (16.6%), which would indicate larger diffuse lung injury, immunosuppression and hypoalbuminemia due to the CPB time longer than 100 minutes, associated with multiple transfusions due to increased bleeding that occurred in these patients. An increased bleeding rate was observed in the conventional surgery group, although no statistical differences were found.

Renal failure occurred in only one patient from the hybrid technique and there was no statistical difference between the groups.

Thus, in relation to the complications, the fact that there were no statistical differences between the groups may be due to the small sample size, since only patients with aortic arch aneurysm not affecting the surrounding anatomical regions were considered. The aortic arch aneurysm alone corresponds to only 10% of the thoracic aorta aneurysms^[3]^.

The mean ICU time was similar in both groups, being 3 days for the hybrid technique group and 2 days for the conventional technique group, with no significant difference.

Melissano et al.^[17]^ reported a mean hospitalization time of 9 days for hybrid treatment in Zone 0; Moulakakis et al.^[1]^ found a mean hospitalization time of 12 days for patients underwent to hybrid surgery. In this study, the mean hospitalization time in patients submitted to the hybrid technique was 8 days.

Some authors have adopted the hybrid surgery as first-line intervention, due to technical advantages like absence of aortic clamping, removal of CPB and hypothermia. But these data were not properly based on the literature, which is contradictory regarding the superiority of the hybrid surgery compared to conventional surgery.

The hybrid surgery has been employed to high-risk patients especially in urgency and emergency contexts. Moreover, lesion morphology, aorta anatomy and comorbidities of the patients ultimately determine the operative risk and prognosis. Therefore, besides the surgical technique, patients' clinical and anatomical conditions are also determinants for the results.

As limitations of this study, it can be emphasized the small sample size, which may have not been enough to detect differences between groups, and a possible bias in the patient selection, since the hybrid surgery tended to be more indicated in higher operative risk patients. It is noteworthy to say that, from 948 patients with aorta aneurysms or dissections in all its segments operated on the MTH during this period, only 25 patients were selected due to the rigid inclusion criteria, which excluded patients who simultaneously presented ascending and/or descending aorta impairment, in addition to cases of acute aortic dissection. This criterion has restricted the sample size, since most patients with aortic arch aneurysm had also affected surrounding segments.

Although a lower mortality rate was expected in the hybrid surgery group, the contrary was found: a higher mortality rate (23%) compared with the mortality rate of the conventional surgery group (17%), but with no significant difference (*P*=0.248). In addition, lower complications rate for the hybrid technique was also expected, which, again, did not occur. Therefore, the hybrid technique did not reveal superiority over the conventional surgery. However, as beforehand mentioned, this may be due to the selection bias.

Hence, the results found in this study did not confirm the hypothesis of superiority of the hybrid technique for the treatment of aortic arch aneurysm. On the other hand, it continues to be an alternative to the conventional surgery, especially in high-risk patients.

## CONCLUSION

The mortality and postoperative complications rates between hybrid and conventional surgery for the aortic arch aneurysms treatment were similar, with no superiority of one technique over the other.

However, due to the small sample, comparative studies with larger samples are needed to elucidate what would be the best option in these cases.

**Table:** 

Authors' roles & responsibilities	
LOS	Substantial contributions to the conception or design of the work; or the acquisition, analysis, or interpretation of data for the work; final approval of the version to be published
RCB	Realization of operations and/or trials; final approval of the version to be published
TPN	Substantial contributions to the conception or design of the work; final approval of the version to be published
RJP	Acquisition, analysis, or interpretation of data for the work; final approval of the version to be published
FARRF	Drafting the work or revising it critically for important intellectual content; final approval of the version to be published
LCML	Drafting the work or revising it critically for important intellectual content; final approval of the version to be published
ELS	Acquisition, analysis, or interpretation of data for the work; final approval of the version to be published
